# Dual Functional Materials: At the Interface of Catalysis and Separations

**DOI:** 10.1021/acs.langmuir.3c03888

**Published:** 2024-03-12

**Authors:** Rashad Ahmadov, Shane S. Michtavy, Marc D. Porosoff

**Affiliations:** Department of Chemical Engineering, University of Rochester, Rochester, New York 14627, United States

## Abstract

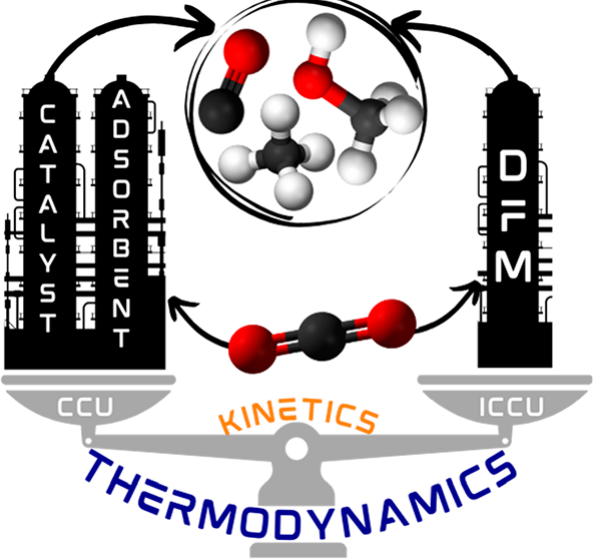

Dual functional materials (DFMs) are a promising approach to increase the energy efficiency of carbon capture and utilization by combining both steps into a single unit operation. In this Perspective, we analyze the challenges and opportunities of integrated carbon capture and utilization (ICCU) via a thermally driven process. We identify three key areas that will facilitate research progress toward industrially viable solutions: (1) selecting appropriate DFM operating conditions; (2) designing and characterizing interfacial site cooperativity for CO_2_ adsorption and hydrogenation; and (3) establishing standards for rigorous and comprehensive data reporting.

## 1 Introduction

Dual functional materials (DFMs) are under investigation for enabling integrated carbon capture and utilization (ICCU), which combines CO_2_ capture and utilization (CCU) into a single unit operation. In this Perspective, we are assessing thermally driven DFMs that are designed for processes where CO_2_ and waste heat are both available, theoretically reducing the energy and infrastructure costs of capturing and converting CO_2_ via process intensification.^1^

**Figure 1:**
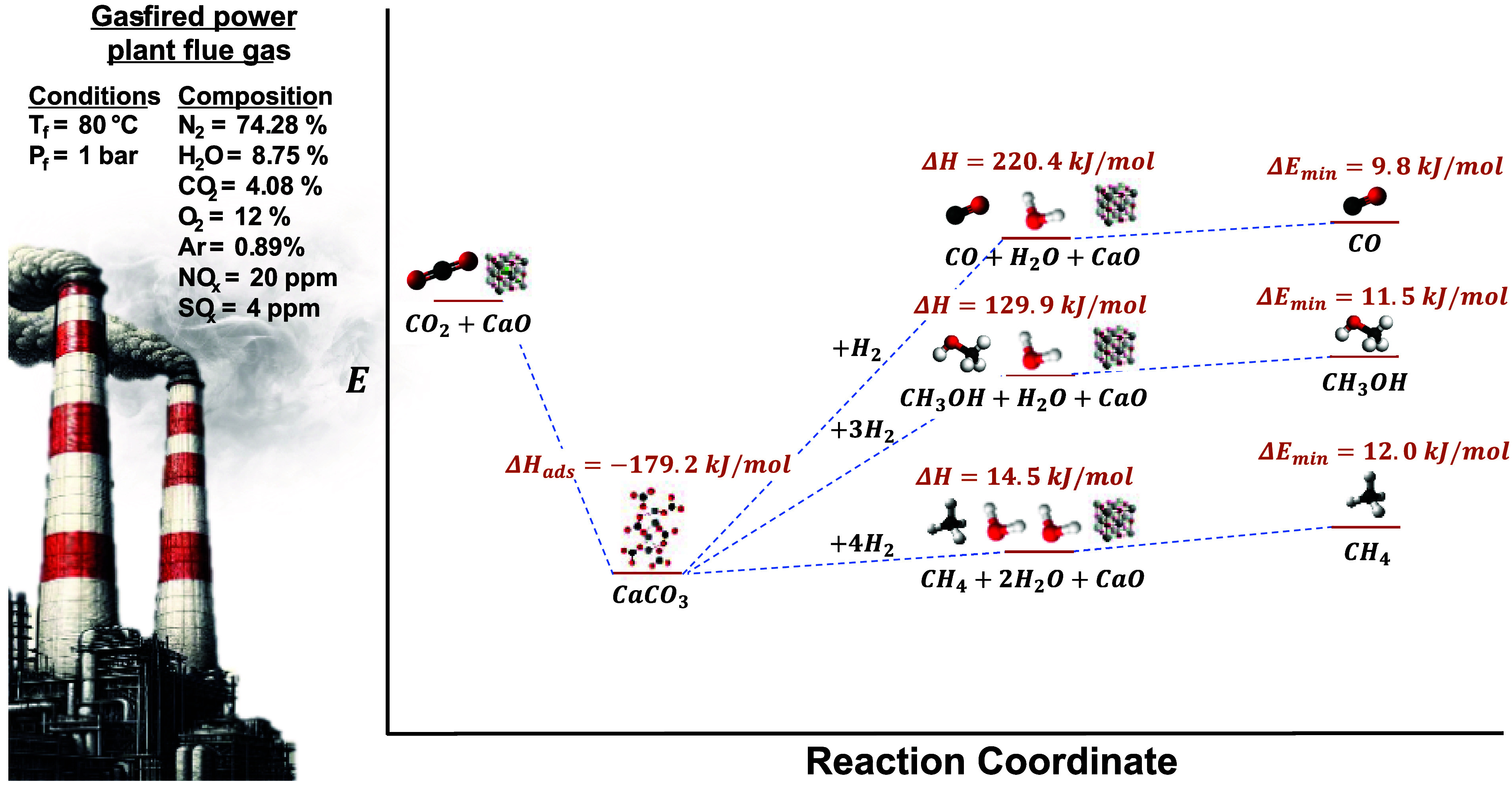
Schematic of commonly studied reactions over DFMs with associated heats of reaction. Right-most thermodynamic state is calculated from the minimum energy required to separate the desired product, assuming 10% conversion of inlet CO_2_.

The underlying principle of DFMs is reactive separation of captured CO_2_ to decrease the number of unit operations needed for CO_2_ utilization. One of the most well-studied approaches uses group IIA metal oxides (*e.g*., CaO) coupled with methanation catalysts (*e.g*., Ru) to capture CO_2_ from flue gas and store excess renewable energy as methane. In the intended process, the methane product is integrated into the existing natural gas infrastructure for consumption during periods of increased energy demand. The DFM framework is particularly attractive because of the perception that flue gas contains sufficient thermal energy to drive CO_2_ desorption and enhance the rate of CO_2_ hydrogenation.^2^ Although methane is the most well-studied product, DFMs have also been explored for producing CO and methanol, as shown in Figure 1. The reactions and associated enthalpies are listed below, with flue gas compositions taken from DOE and EPA reports.^3,4^ There are examples in the literature of reducing the bound CO_2_ with methane, but these studies are mechanistically more complex and outside the scope of this Perspective.^5^

1. Methane (Sabatier):
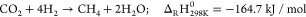


2. Carbon monoxide (reverse water–gas shift):
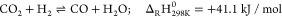


3. Methanol:
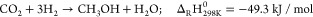


The overall ICCU process consists of three primary steps: (1) *CO*_2_*adsorption:* CO_2_-rich flue gas is flowed over the DFM, and CO_2_ binds to the adsorbent. (2) *Inert purge:* An inert gas is flowed over the DFM to remove O_2_ from the system, an important consideration for safe operation. (3) *Reactive separation:* Hydrogen is introduced to react with the bound CO_2_ to facilitate desorption and produce value-added products.^6,7^ Importantly, the thermodynamics for ICCU-related reactions are no different than those of the overall CO_2_ hydrogenation reactions. The key distinction between the two processes is that CO_2_ is reactively separated from flue gas in one step instead of separating and reacting CO_2_ over two different materials in separate locations. In this Perspective, we are focused on CO_2_ capture and conversion from flue gas because of the high entropy penalty of separating CO_2_ from air and significant thermal losses that may occur when DFMs are used during direct air capture (DAC) if the residual heat after reaction is not efficiently utilized.^8^ The efficiency of capturing CO_2_ as a function of the source concentration is detailed in the Sherwood plot in Figure S1 of the Supporting Information.^9^

To accelerate progress in DFM research, we need to ensure that DFMs are studied under the relevant conditions, without the pretense that a better DFM will solve all implementation challenges. For example, DFM mechanisms are controversial, with studies suggesting both Langmuir–Hinshelwood and Eley–Rideal kinetics.^10^ Part of this discrepancy is likely due to intrinsic kinetics, which is expected for different adsorbents and catalysts. However, mechanistic and performance discrepancies may also be a result of inadequate benchmarking and inconsistencies in operating conditions and data reporting.

If we use Ru/K_2_CO_3_-MgO as an example DFM for CO_2_ methanation, under the best conditions approximately 1.12 mmol of CH_4_ is produced per gram of DFM.^11^ At the studied loading of 0.5 g of DFM, 0.56 mmol of CH_4_ is produced per capture and conversion cycle. During the hydrogenation step, 40 mL/min of 90% H_2_ is flowed over the DFM for 1 h, which equals a total of 88.5 mmol H_2_ per cycle at 25 °C and 1 atm. To produce the 0.56 mmol of CH_4_, 2.24 mmol of H_2_ is consumed, meaning that if we assume full conversion of CO_2_, the ratio of species in the reactor outlet (neglecting N_2_) is 158/1/2 H_2_/CH_4_/H_2_O, or *0.62% CH*_4_, *which is an order of magnitude lower than the concentration of CO*_2_*in flue gas*. This is an important and often overlooked consideration because decreasing the amount of H_2_ during the hydrogenation step will make the reaction thermodynamics and kinetics less favorable, whereas high H_2_/CO_2_ ratios are not industrially viable because renewable H_2_ is wasted and/or additional energy is needed to recover the desired product.

In this Perspective, we seek to bring attention to more nuanced considerations of DFM design and implementation that are not adequately addressed in the literature. We outline some of the outstanding questions in the thermal DFM space to encourage targeted kinetic studies that clearly report reaction conditions and performance metrics to accelerate DFM advancement and implementation. The subsequent sections focus on the following questions:

1. *Are there specific conditions where ICCU will outperform CCU?*
2. *What are the current challenges of advancing DFM basic science?*
3. *How can the performance of new DFMs be effectively assessed and reported?*

## 2 DFMs: Challenges and Opportunities

### 2.1 Investigating the Ideal Conditions for Integrated Carbon Capture and Utilization

ICCU is a potentially energy efficient alternative to traditional multi-unit CCU. This is because ICCU is simplified, without any need to concentrate and transport CO_2_ prior to the hydrogenation step, relying on the principle that heat from exothermic CO_2_ adsorption is efficiently utilized to operate the process. There is some debate between operating the process isothermally or with a temperature swing because of trade-offs that are difficult to quantify. Temperature swings improve CO_2_ capture efficiencies and product yield because CO_2_ binding is thermodynamically favorable at lower temperatures.^11^ However, temperature swings are a parasitic energy load on the process, which also degrade CO_2_ adsorbents and hydrogenation catalysts.^1^

It is not completely clear how DFMs would enable an increased efficiency for ICCU via process intensification. There have been some recent techno-economic analyses (TEA) on DFMs, but the reports do not provide adequate information to be reproduced in Aspen, so it is difficult to know how sensitive the models are to variations in energy sources, flue gas contaminants, and downstream separations.^2,12^ It is also challenging to determine which products are more practical or desirable, but this decision likely depends on the selected CO_2_ source, and difficult to predict process variables. For example, in CaO-based DFMs, exothermic CO_2_ adsorption releases 178.2 kJ/mol, and TEA analysis suggests this heat must be recovered for the process to be economical, specifically if the desired product is CO via endothermic reverse water-gas shift (RWGS; Δ_R_H^0^_298K_ = +41.1 kJ/mol).^2^

If methane is the desired product, the process may benefit from heat recovery because the reaction exotherm of 164.7 kJ/mol can be used to facilitate both CO_2_ desorption and DFM heating to the reaction temperature of *ca*. 300 °C. However, even if we assume ideal utilization of waste heat, the energy calculus of ICCU with CO_2_ methanation becomes less favorable when we consider the H_2_ lifecycle.

Careful inspection of the reaction equations shows that twice as much H_2_ is needed for water formation during CO_2_ methanation relative to RWGS, meaning there is an effective parasitic energy load on the process of 285 kJ/mol that is expended during water electrolysis. Even though we gain 164.7 kJ/mol thermal energy during CO_2_ methanation, we must expend an additional 285 kJ/mol relative to that of RWGS to produce sufficient H_2_ for water formation, negating any energy savings from heat recovery during CO_2_ methanation.

It is also important to consider the downstream separation when comparing the viability of the products. H_2_ is typically used in excess to achieve high CO_2_ conversions and CH_4_ selectivities that exceed 90% on a basis of adsorbed CO_2_.^11^ However, thermodynamics prevents CO_2_ methanation from operating at full conversion, indicating that renewable H_2_ will be wasted in the product stream, which is undesirable because the purpose of the process is to store renewable energy as CH_4_.^13^ If significant amounts of H_2_ are mixed with CH_4_ at the reactor outlet, then the overall scheme of hydrogenating CO_2_ becomes less efficient than using H_2_ directly.

Targeting CO or methanol instead of CH_4_ might offer more flexibility in the overall ICCU process. For CO, a CO/H_2_ separation is not needed because the amount of unreacted H_2_ can be tuned to control downstream Fischer–Tropsch synthesis.^14^ However, the higher reaction temperature of 650 °C that is often used for RWGS will decrease the overall process efficiency because of the need to extract additional thermal energy, especially when reheating flue gas during isothermal operation.^15^ There are some efforts that seek to take advantage of fluctuating feed compositions by using switchable DFMs, where the product (CO or CH_4_) can be tuned with the DFM operating conditions.^16^

Methanol is another intriguing product because of the lower temperature differences between flue gas (*ca*. 100 °C) and the desired reaction conditions (*ca*. 250 °C). Methanol is also easier to transport because it is a liquid at room temperature, and a recent report indicates that the technology is feasible, although the pressure mismatch between flue gas (1 bar) and methanol synthesis (50–100 bar) is a major challenge.^17^

DFMs are an exciting area of research because we need to design improved materials that will enable economical processes to encourage CO_2_ utilization. By first developing a better understanding of the realistic conditions under which DFMs will be operated, we equip ourselves for benchmarking studies aimed at designing DFMs to kinetically couple CO_2_ desorption with hydrogenation.

### 2.2 Advancing DFM Basic Science

Kinetic studies are a key aspect of understanding DFM performance to better predict the behavior during scale-up. One important challenge is designing materials that operate with high working capacities at high temperature to maximize the CO_2_ available for reaction. We also need to identify catalysts that achieve fast kinetics to ensure the reaction proceeds at a faster rate than CO_2_ desorption, thereby minimizing CO_2_ leakage during purge and reaction.^3,18^ DFMs are a difficult field to advance because we need to develop an improved understanding of the adsorbent, catalyst, and adsorbent–catalyst interface for enhanced DFM kinetics.

DFMs operate at much higher temperatures (*ca*. 300 °C) than typical amine-based CO_2_ scrubbing technology (*ca*. 80 °C) because additional thermal energy is needed for fast CO_2_ hydrogenation kinetics.^3,19^ Group I and II metal oxides are commonly studied as the adsorbent component of a DFM due to their high reactivity toward CO_2_ and relatively low cost. There have been some instances of using CaO as a stand-alone DFM at 600–700 °C, but, sintering is a challenge because CaO expends when carbonated to CaCO_3_, which can mask the catalyst surface and reduce capture capacity.^20^ To mitigate CaO sintering, some studies use MgO, Y_2_O_3_ and Al_2_O_3_ as stabilizers, which act as a physical barrier between neighboring CaO particles, thereby preventing agglomeration over multiple adsorption–reaction cycles.^15,21,22^ In one example, adding 11 wt % MgO to CaO leads to a CO_2_ uptake that exceeds the CaO reference by 5× after 30 cycles.^21^

CO_2_ uptake is not the only indicator of DFM performance; kinetic parameters are just as important for DFM implementation. We illustrate this point with a natural gas combined cycle (NGCC) plant, which produces approximately 4 million kilograms of flue gas per hour.^3^ If we achieve a 10% CO_2_ capture efficiency, the target CO_2_ capture amount is 575 kmol/h. With a calculated flue gas molecular weight of 28.4 kg/kmol and a cycle time of 1 h with a target adsorption capacity of 5 mmol CO_2_/g DFM, we require approximately *115 t of DFM*. If we shorten the cycle time to 15 min and use a DFM with a capacity of 10 mmol CO_2_/g, the required amount of DFM decreases by a factor of 8 to *14.4 t*, illustrating that we need DFMs with both high CO_2_ adsorption capacity *and* fast adsorption/reaction kinetics.

**Figure 2:**
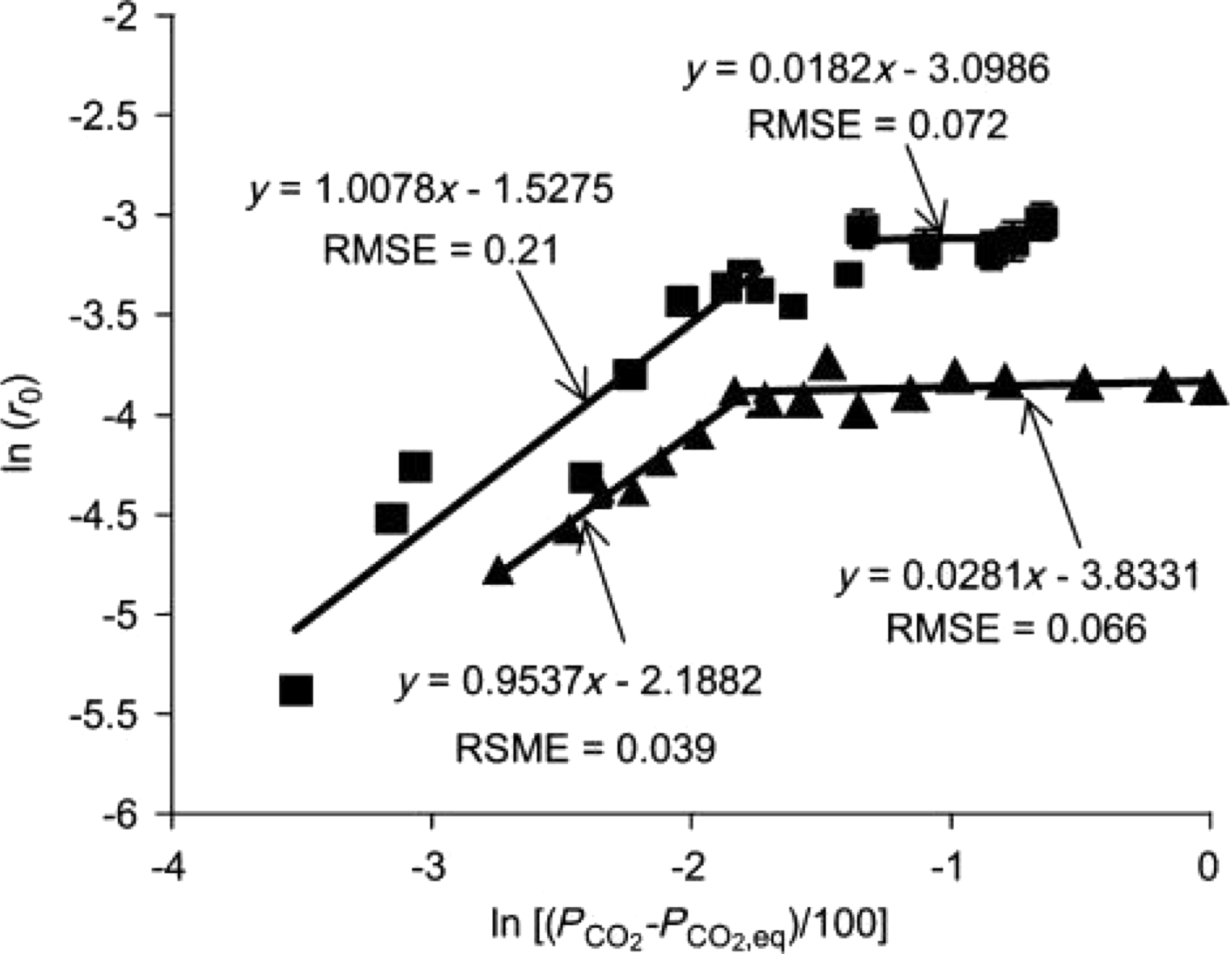
Transition of the adsorption mechanism as a function of CO_2_ partial pressure over CaO. For high CO_2_ partial pressures the carbonation reaction is 1st order in CO_2_. On the other hand, for a low CO_2_ partial pressure the carbonation reaction becomes 0th order in CO_2_. Reprinted with permission from ref (23). Copyright 2013 WILEY-VCH Verlag GmbH & Co. KGaA, Weinheim.

Decreasing the DFM cycle time requires a better understanding of the kinetic parameters, as illustrated in Figure 2. As shown in the figure, CO_2_ adsorption kinetics exhibit Langmuir isotherm behavior with a transition from first to zeroth order kinetics as a function of the CO_2_ partial pressure. At low CO_2_ partial pressures, CO_2_ is a limiting reactant, but at partial pressures of *ca*. 10 kPa, CaCO_3_ formation becomes rate limiting, leading to zeroth order kinetics with respect to CO_2_.^23^ These findings are extremely relevant to DFM studies because the transition from first to zeroth order occurs at *ca*. 10 kPa CO_2_, or 10% CO_2_ at atmospheric pressure, a commonly studied CO_2_ concentration.^2,11,16,24^ The exact transition likely shifts as a function of the DFM identity, but this study suggests that it may be difficult to compare DFM performance because the adsorption kinetics transition is close to commonly studied conditions.

**Figure 3:**
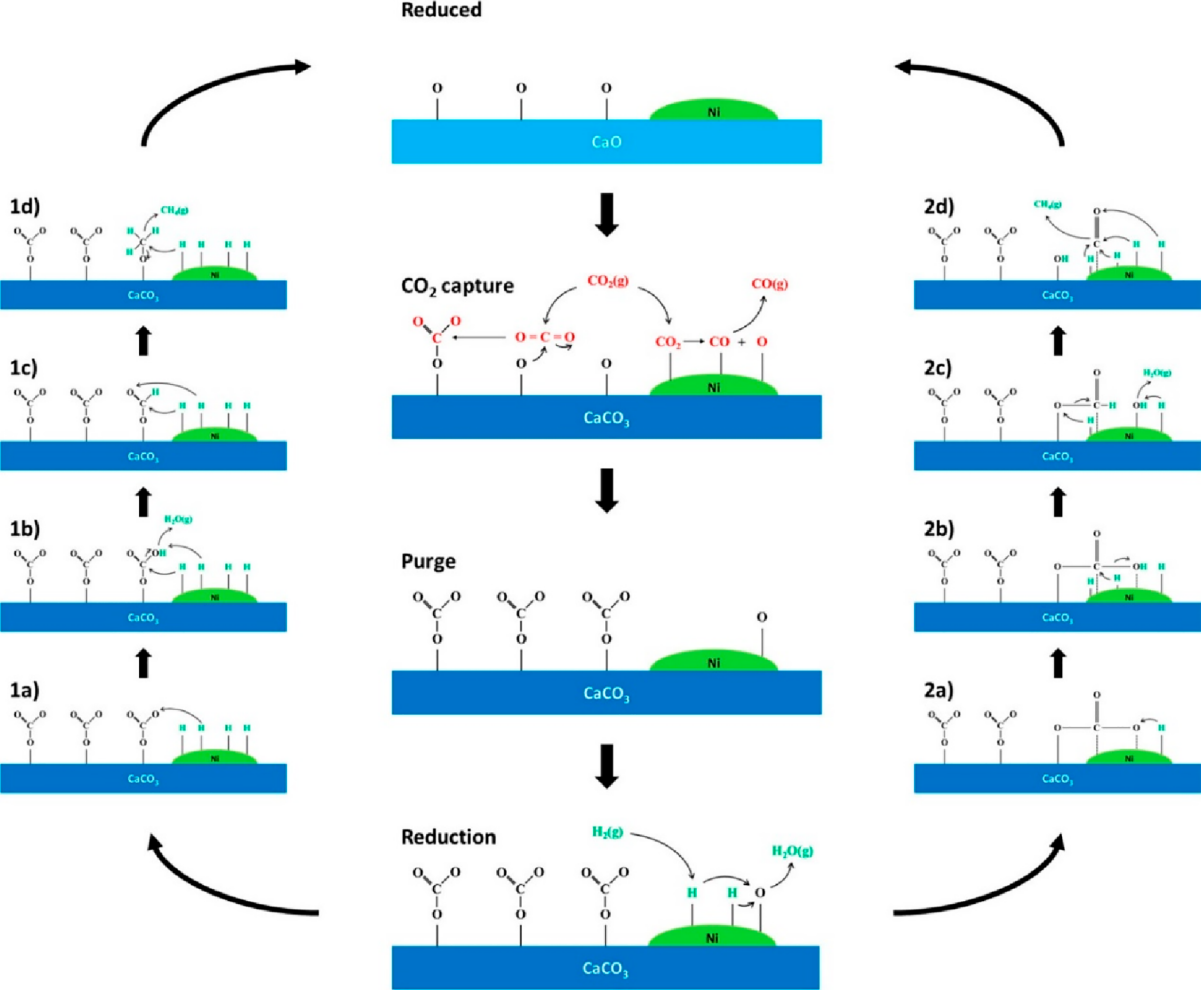
Proposed integrated CO_2_ capture and direct methanation over Ni/CaO-based DFMs. Reprinted with permission from ref (25). Copyright 2023 American Chemical Society.

For the catalyst component, nickel-based DFMs are commonly studied because of their low cost and high performance in the absence of steam and oxygen. The mechanism of CO_2_ capture and conversion over Zr-modified Ni/CaO-based DFMs has been studied extensively with *in situ* Fourier transform infrared spectroscopy and is illustrated in Figure 3.^25^ During the CO_2_ adsorption step, the carbon atom of CO_2_ acts as a Lewis acid, which binds strongly with the O^2–^ ion from CaO to form monodentate carbonates and CaCO_3_. The CO_2_ molecule can also dissociate on the metallic Ni surface to form CO* and O* to produce NiO and gas phase CO. During the hydrogenation step, there are two proposed routes for CH_4_ formation: (1) Adsorbed monodentate carbonate is hydrogenated through a series of intermediates: bicarbonate, formate, methoxy, and methane. (2) Carbonates spill over onto the Ni surface and react with Ni hydrides to form bicarbonates. The bicarbonates are then dehydrated to formate and then hydrogenated to carbonyls and ultimately CH_4_ at the reaction temperature of 500 °C.

Designing an effective DFM also requires consideration of the synergy between the adsorbent and the catalytic active site, as illustrated in Figure 3.^25^ There have not been many studies on the spatial arrangement of the two components, but a recent report hypothesizes that the proximity between the CO_2_ adsorbent and hydrogenation catalyst is critically important for controlling the reaction kinetics.^26^ Similar phenomena are observed in tandem reactions in heterogeneous catalysis, where the diffusion distance of intermediates affects the reaction rate and product selectivity.^27^ Localized heat transfer may also be important to consider and is an intriguing opportunity for further study. We hypothesize that the top performing DFMs use local thermal gradients during exothermic hydrogenation to facilitate desorption and reaction.^28^

Kinetic studies become more complicated when common flue gas contaminants are included in the experiments. Although simplified kinetic studies with clean and dry flue gas are helpful for measuring intrinsic kinetics, using more realistic simulated flue gas will help bridge the gap between academic and industrial studies, accelerating DFM implementation.^29^ The most common components of flue gas from a natural gas power plant other than CO_2_ (4.08%) and N_2_ (74.28%) are SO_*x*_ (4 ppm), NO_*x*_ (20 ppm), oxygen (12%), water (8.75%), and argon (0.89%).^3,4^ For the concentrations of minor contaminants (SO_*x*_ and NO_*x*_), it is difficult to find reliable data in the literature, so our values are set at the maximum permissible limit set by the U.S. Environmental Protection Agency.^4^ Studying flue gas in the presence of contaminants is important because they generally have negative effects on DFM performance. SO_*x*_ diminishes CO_2_ capture capacity and methane production by *ca*. 75%,^30^ NO_*x*_ competitively adsorbs with CO_2_ on basic sites,^31^ and oxygen oxidizes active sites. The effect of water is complex and depends on the DFM composition.^32^

There have been detailed studies that have focused on mitigating the negative effects of oxygen and water. Ni-based DFMs are particularly prone to oxidation when the reaction temperature is below the temperature required to reduce NiO to metallic Ni.^33^ To prevent deactivation, small amounts of precious metals are added to Ni-based DFMs to facilitate NiO reduction under reaction conditions, a practical solution to maintain a relatively low DFM cost during scale-up.^34^

The effect of water is complex, with studies showing that water enhances CaO reactivity with CO_2_, removes coke, and increases pore size, leading to faster diffusion and carbonation.^3^ However, during calcination, steam promotes sintering, which decreases the available sites for adsorption, and over many cycles the presence of steam tends to reduce CO_2_ capture capacity.^32^ An important area of future research is identifying straightforward methods of increasing DFM tolerance to the commonly studied contaminants in flue gas, particularly to those that are not present during typical CO_2_ hydrogenation experiments.^35^

### 2.3 Challenges of DFM Implementation

Advancing DFM science and implementing solutions for carbon capture and utilization require clear reporting to facilitate benchmarking. Evaluating and comparing the performance of DFMs is hindered by inconsistencies in the literature on gas composition, gas hourly space velocity (GHSV), purging, regeneration, and particle size.^10,16^ Without well-defined benchmarks and clearly delineated experimental conditions, it is difficult to extract performance trends and design improved DFMs for the ICCU.

A significant challenge in the field is that we lack the data to thoroughly understand the effects of heat and mass transfer limitations during DFM experiments. For example, the concentration of CO_2_ affects the rate of CO_2_ diffusion and, in turn, adsorption onto the DFM surface. Research groups use a range of compositions in the CO_2_-rich gas stream, spanning 400 ppm for direct air capture to 17% for simulated flue gas, as shown in Table 1.^17,24,36^ The CO_2_ concentrations are selected to simulate the end-use application, but it is generally not discussed if a higher CO_2_ concentration leads to localized exotherms, which in turn decreases the overall rate of adsorption. Similar arguments can be made for the H_2_ concentration during the reaction step, where the significance of heat transfer limitations is not well-studied, particularly as a function of the H_2_ concentration and reaction rate.

**Table 1:** Summary of Selected DFM Performance To Highlight the Range of Testing Conditions^a^

	**CO**_**2**_**capture**			**CO**_**2**_**hydrogenation**			
**DFM**	**Feed (%)**	**T (°C)**	**GHSV(mL/h/g)**	**Reductant (%)**	**T (°C)**	**GHSV(mL/h/g)**	**Cycle time (min)**
**Methanation**							
Ni-Ru-CaO/CeO_2_-Al_2_O_3_^16^	10CO_2_/90N_2_	350	12000	10H_2_/90N_2_	350	12000	20
Ru-CaO/γ-Al_2_O_3_^24^	10CO_2_/90 dry air	320	1043	5H_2_/95N_2_	320	5365	20
Ru-CaO/γ-Al_2_O_3_^24^	8CO_2_/21H_2_O/71 air	320	1282	5H_2_/95N_2_	320	5250	20
Ru-CaO/Al_2_O_3_^32^	7.5CO_2_/15H_2_O/4.5O_2_/73N_2_	320	11236 h^–1^	5H_2_/95N_2_	320	2611 h^–1^	20
Ru/K_2_CO_3_-MgO^11^	10CO_2_/10H_2_O/80N_2_	150	4800	90H_2_/10N_2_	320	4800	outlet = inlet
Ni-Na_2_O/Al_2_O_3_^33^	7.5CO_2_/4.5O_2_/15H_2_O/73N_2_	320	4000 h^–1^	15H_2_/85N_2_	320	8000 h^–1^	80
Li-Ru/Al_2_O_3_^37^	5CO_2_/0.25O_2_/0.01SO_2_/94.75N_2_	260	21786	15H_2_/85N_2_	260	33000 h^–1^	32
Ru-Na_2_CO_3_/Al_2_O_3_^36^	5CO_2_/95N_2_	320	18000	4H_2_/96N_2_	320	15600	150
Ni/CaO-Zr^25^	10CO_2_/10H_2_O/80N_2_	500	4800	90H_2_/10N_2_	500	4800	N/A
Ru-Na_2_CO_3_/Al_2_O_3_^11^	0.04CO_2_/air	25	2000	15H_2_/85N_2_	500	12000	>360
**Reverse water-gas shift**							
Ni-Ru-CaO/CeO_2_-Al_2_O_3_^16^	10CO_2_/90N_2_	650	12000	10H_2_/90N_2_	650	12000	20
Fe_5_Co_5_MgO_10_CaO^2^	10CO_2_/90N_2_	650	5730 h^–1^	100H_2_	650	5730 h^–1^	62
CeO_2_-CaO^38^	17CO_2_/83N_2_	650	20000	5H_2_/95N_2_	650	20000	53
Ni-CaO-CeO_2_^39^	10CO_2_/90N_2_	650	120000	10H_2_/90N_2_	650	120000	30
Ca_1_Ni_0.1_Ce_0.033_^15^	15CO_2_/85N_2_	650	24000	5H_2_/95N_2_	650	24000	95
La-Ni/Ca^40^	10CO_2_/90Ar	650	6000	10H_2_/Ar	650	6000	140
Ni/CaO^41^	10CO_2_/10H_2_O/80N_2_	700	12000	10H_2_/90N_2_	700	12000	310
**Methanol synthesis**							
K/Cu-Zn-Al^17^	1CO_2_/99He	100	6000	100H_2_	250	6000	180

a We have normalized GHSV to units of mL/h/g or h^–1^ for ease of comparison.

The balance gas might also play a role as the primary mechanism for heat removal from the DFM interface. DFM studies use one of three balance gases, N_2_, Ar, or He. It is not clear whether the thermal conductivity differences among N_2_ (0.026 W/(m K)), Ar (0.018 W/(m K)), and He (0.151 W/(m K)) are significant enough to convolute DFM kinetic measurements. Perhaps it is possible that the order of magnitude greater thermal conductivity of He could enhance the CO_2_ adsorption/desorption kinetics because of faster interfacial heat transfer in the CO_2_/He system relative to CO_2_/N_2_. These effects could also be decreased by diluting the DFM bed with silicon carbide to reduce heat transfer limitations.

Mass transfer limitations have been studied to a greater degree than heat transfer limitations,^32^ but understanding DFM performance data in context is difficult when adsorption studies are conducted at a wide range of GHSVs from *ca*. 1,000 to 120,000 mL/g/h in Table 1. To facilitate comparison for the reader, we have standardized the units of GHSV to illustrate that some testing conditions may be limited by mass transfer, leading to conclusions that could be misleading by not encapsulating the intrinsic DFM performance. We expect low GHSVs to exhibit significant mass transfer limitations because of an increased boundary layer around the DFM particles, leading to a diffusion-controlled process.

Interpreting DFM performance is challenging because researchers seek to report the efficacy of DFM in both CO_2_ capture and conversion. The effort becomes more challenging when the performance metrics themselves are inconsistent or reliant on GHSV or cycle time for appropriate context. For example, representing DFM performance via mass of product per mass of DFM does not include any kinetic parameters and could be biased by a longer cycle time.^11^

There is also some confusion with conversion calculations, because they are inconsistent within the DFM literature and differ from the typical reaction used in catalysis. The discrepancy between the calculations ultimately depends on the basis of the calculation: CO_2_ flowed over the DFM, CO_2_ adsorbed, or CO_2_ desorbed plus products formed.^11,37^ Selected definitions from the literature are summarized below:
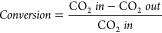
1
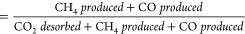
2
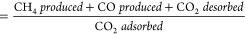
3

DFM studies do not typically use the catalysis definition of conversion and instead define “CO_2_ in” as either “CO_2_ adsorbed” or “CO_2_ desorbed plus products”, an approach that is more prone to experimental error, inflates the actual amount of CO_2_ converted by the DFM, and is insensitive to cycle time. For “CO_2_ adsorbed”, the calculation is more prone to error than definition (1), because it is dependent on first measuring the amount of CO_2_ adsorbed instead of using the known flow rate of CO_2_ into the reactor. In the case of “CO_2_ desorbed”, it is likely that a fraction of the adsorbed CO_2_ does not desorb from the DFM, which is mistakenly mathematically counted as converted CO_2_, thereby inflating the conversion calculation.^11^

The two commonly used definitions are also not a function of the cycle time, which is important because if 10% CO_2_ is flowed over 0.5 g of DFM at 40 mL/min for 1 h, 1.12 mmol CH_4_/g DFM translates to *an actual CH*_*4*_*yield of 5.7%*, and not 97.4% when 1.15 mmol CO_2_ captured/g DFM is used as the basis of the calculation.^11^ Furthermore, using definitions (2) or (3) makes it difficult to calculate the maximum equilibrium conversion because the equilibrium value is a function of the H_2_ amount flowed over the DFM, which is generally orders of magnitude larger than the stoichiometric amount, as we show in the Introduction where the effluent H_2_/CH_4_ ratio is 158/1. With such a large amount of H_2_ in the system, the thermodynamic equilibrium is shifted heavily toward products, rendering the widely-adapted DFM definitions of conversion meaningless. We clearly need better benchmarks and more consistent reporting to advance DFM science and push the field toward faster kinetics and rates of CO_2_ capture and conversion.

## 3 Pathways for Accelerating DFM Progress

Within the scope of this Perspective, we have highlighted three key areas that require improvement to advance DFM science: (1) understanding the ideal conditions for integrated carbon capture and utilization; (2) developing an improved understanding of DFM kinetics and adsorbent/catalyst interactions; and (3) establishing standards for improved benchmarking and data reporting. In these three broad areas, we have elucidated a few common themes, along with specific recommendations and directions for future research.

### 3.1 DFM Performance Metrics

Without clear performance metrics and benchmarks, it will be difficult to advance DFM science. In this Perspective, we discuss some of the challenges and inconsistencies associated with H_2_ amounts, GHSVs, and conversion calculations. Although we have identified instances of different definitions of conversion, it is rare to find details of H_2_ conversion in the literature, which is important for providing context to how much H_2_ is consumed during the DFM experiments.^42^ It is also worth noting that it is not atypical, as shown in Table 1, that the GHSV during hydrogenation is different from that during CO_2_ capture. This discrepancy can be confusing for readers and again makes it challenging for scientists in the field to use their intuition to contextualize DFM performance without computational assistance.

We strongly recommend that researchers change their CO_2_ conversion metric to use the same standard as that for catalysis, which uses CO_2_ molar flow rate at the inlet as the basis of the calculation. This new convention would benefit the field for a variety of reasons: (1) Simpler to calculate with less error because it is not reliant on calculating CO_2_ adsorbed or desorbed–the CO_2_ flowed into the system is a known quantity; (2) consistent definition that makes results easier to contextualize; and (3) the catalysis convention encapsulates both CO_2_ adsorption and conversion in a single value, which is a key indicator of DFM performance. We recognize that established researchers in the DFM field may be resistant to adapting our proposed definition because conversion values will be deflated relative to previous calculations that use “CO_2_ adsorbed” or “CO_2_ desorbed plus products” as the basis of the calculation.^43^

### 3.2 Matching the Product with the Process

Product selection over DFMs should not be arbitrary and is a strong function of the process in which ICCU will be implemented. Using flue gas from coal fired power plants is likely not feasible because of the high particulate matter and sulfur that degrades DFM performance.^3^ NGCC plants are more feasible because sulfur is removed prior to gas combustion, particulate matter is significantly lower than that of coal-fired plants, and the NGCC plants are already equipped with heat recovery systems that may be compatible with DFM technology.^3,4^

From our analysis of the literature, it is not completely clear how much heat is available for DFM operation, but the simplest case would be to implement the DFM at the flue gas stack, where the temperature is 80–100 °C.^3^ These temperatures are too low for CO_2_ hydrogenation, indicating that there is a need for a detailed Aspen study that reports all parameters and considers practical levels of heat recovery to accurately determine the costs associated with running ICCU at a NGCC plant. It is also necessary to run a full sensitivity analysis with H_2_ production and product separation because it is currently challenging to compare the energy and economic cost of each targeted product.

### 3.3 Mechanistic Studies

Additional mechanistic studies over DFMs that utilize *in situ* and/or operando techniques are needed to better understand DFM performance.^25,44^ The field is ripe for deeper analysis that uses advanced techniques, including *in situ* X-ray absorption spectroscopy and environmental transmission electron microscopy, to gain more insight into the DFM structure under reaction conditions. As discussed in this Perspective, there are some kinetic studies that analyze proximity effects, but we need more insight into heat and mass transfer limitations to better tune DFM performance.^26^ This is particularly the case for CO_2_ methanation, where it is not clear how CO_2_ desorbs from the surface. There are studies that report CO_2_ is hydrogenated through a spillover mechanism,^24^ but more detail is needed to design improved DFMs.

Some important questions to address regarding DFM mechanisms are the following: (1) How does CaCO_3_ decompose into CaO and adsorbed CO_2_? (2) What provides the driving force for product desorption? Although CO_2_ desorption and hydrogenation are coupled into a single step, thermodynamics requires that sufficient energy is put into the system to desorb bound CO_2_. This energy could originate from ambient thermal energy if the system is held at 300 °C but may also originate from water readsorption or the reaction exotherm. (3) Are there other materials, such as zeolites or metal organic frameworks, that exhibit improved performance relative to the group IIA metal oxides that are commonly used in the DFM field? Advancing DFM science requires more fundamental studies over model systems to elucidate mechanistic features and the difficult to understand nuances of the reaction mechanism.

## 4 Conclusion

The DFM field is relatively new, with most papers published after 2015. There have been fewer than ten years of research to establish benchmarks and clear research directions to help methodically advance DFM science. Although there are many important areas to investigate, including benchmarking, basic DFM science, and data reporting, the promise of DFMs as a technology to decrease the energy demand of CO_2_ capture and conversion is ripe for new research directions that accelerate CO_2_ utilization.

We recommend that researchers who are interested in exploring DFMs carefully consider their experimental conditions to ensure that studies are conducted in kinetically controlled regimes with inlet gas stream compositions that are relevant to industrial applications. Additionally, it is important to mindfully consider the basis of conversion and yield calculations to accurately assess DFM performance, enabling comparisons between studies performed by different research groups. DFMs have high unexplored potential, and it is important that we carefully consider and understand the basic science and principles of this technology to accelerate future deployment and commercialization.

## Supporting Information Available

Sherwood plot cost estimates and minimum work for CO_2_ separation as a function of concentration; calculations for CH_4_ outlet concentration, required DFM amounts, CH_4_ yield using CO_2_ captured versus inlet CO_2_, and minimum energy for CO_2_ separation (PDF)
