# Supplementary material for: Dual Functional Materials: At the Interface of Catalysis and Separations
Source: Langmuir. 2024 Mar 12;40(19):9833–41. doi: 10.1021/acs.langmuir.3c03888 (PMC11100017; doi:10.1021/acs.langmuir.3c03888)
Supplement: Supplementary file 1 — la3c03888_si_001.pdf [file la3c03888_si_001.pdf]

Supporting Information

Dual functional materials: At the interface of catalysis and separations

Rashad Ahmadov[‡], Shane Michtavy[‡], Marc D. Porosoff*

Department of Chemical Engineering, University of Rochester, Rochester, NY 14627, USA

[‡]These authors contributed equally to this work.

*Corresponding author

Table of Contents

Figure S1: Sherwood plot cost estimates and minimum work for separation as a function of CO_2 source concentration 2

Calculation 1: CH_4 outlet ratio at 1.12 mmol CH_4/g_{DFM} 2

Calculation 2: Required amount of DFM at target capacity of 5 mmol CO_2/g DFM 2

Calculation 3: Using CO_2 captured vs. inlet CO_2 as methane yield basis 3

Calculation 4: Minimum energy required for CO_2 separation 3

Figure S1: Sherwood plot cost estimates and minimum work for separation as a function of CO_2 source concentration

Data is from Bains et al.[1,2]

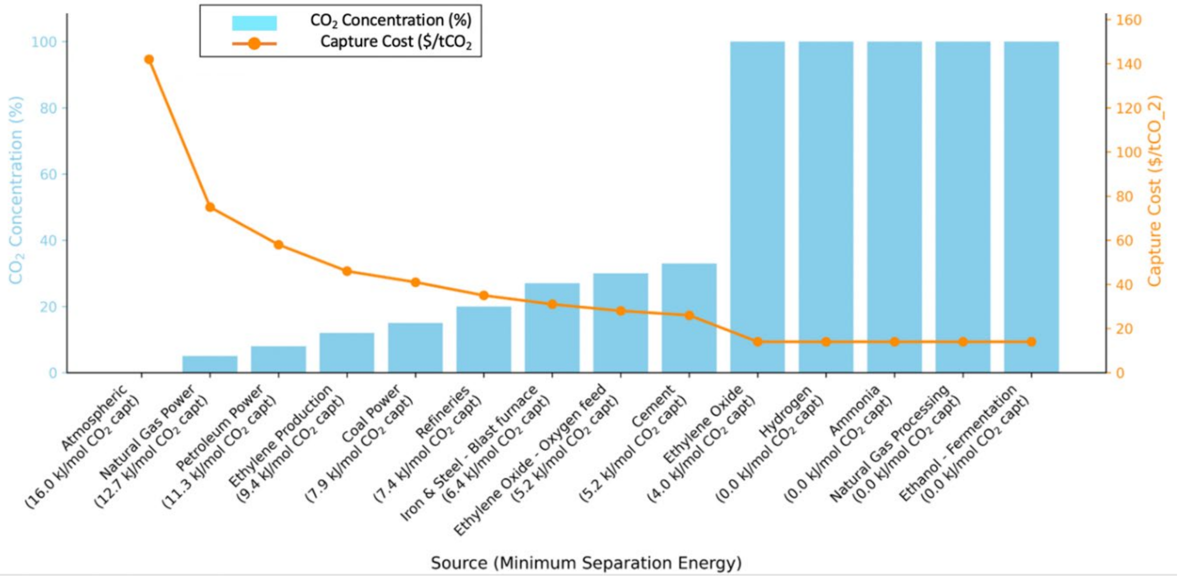

Calculation 1: CH_4 outlet ratio at 1.12 mmol CH_4/g_{DFM}

$$\boldsymbol{Total\ H_2\ per\ cycle};\ \dot{V} = \frac{40\ mL/\min\cdot 60\ mins \cdot 90\%}{24400\ mL/mol} = \mathbf{88.5\ mmol}$$

$$\boldsymbol{Outlet\ H_2{:}CH_4\ ratio};\ \frac{88.5\ mmol\ H_2\ per\ cycle}{0.56\ mmol\ CH_4\ per\ cycle} = \mathbf{158\ H_2 : 1\ CH_4}$$

$$\boldsymbol{CH_4\ composition};\ \frac{1\ mol\ CH_4}{158\ mol\ H_2 + 1\ mol\ CH_4 + 1\ mol\ H_2O} = \mathbf{0.62\%}$$

Calculation 2: Required amount of DFM at target capacity of 5 mmol CO_2/g DFM

$$\boldsymbol{Target\ CO_2\ amount};\ 4\cdot 10^6\ \frac{kg\ flue\ gas}{hr}\cdot\frac{1\ kmol}{28.4\ kg\ flue\ gas}\cdot 4.08\%\cdot 10\% = \mathbf{575\frac{kmol}{hr}}$$

$$\boldsymbol{DFM\ Needed};\ 575\ \frac{kmol\ CO_2}{hr}\cdot\frac{1\ ton\ DFM}{5\ kmol\ CO_2} = \mathbf{115\ tons\ DFM}$$

Calculation 3: Using CO_2 captured vs. inlet CO_2 as methane yield basis

$$\boldsymbol{Total\ CO_2\ per\ cycle\ per\ gram};\ \dot{V} = \frac{40\ mL/\min \cdot 60\ mins \cdot 10\%}{24400\,\frac{mL}{mol} \cdot 0.5\ g\ DFM} = \mathbf{19.7}\ \frac{\boldsymbol{mmol\ CO_2}}{\boldsymbol{g\ DFM}}$$

$$\boldsymbol{CH_4\ yield, CO_2\ captured\ basis};\frac{1.12\,\frac{mmol\ CH_4}{g\ DFM}}{1.15\,\frac{mmol\ CO_2}{g\ DFM}} = \mathbf{97.4\%}$$

$$\boldsymbol{CH_4\ yield, CO_2\ inlet\ basis};\frac{1.12\,\frac{mmol\ CH_4}{g\ DFM}}{19.7\,\frac{mmol\ CO_2}{g\ DFM}} = \mathbf{5.7\%}$$

Calculation 4: Minimum energy required for CO_2 separation

Neglecting the enthalpy of mixing, the minimum work required to separate CO_2 from air is the reverse of the entropy of mixing.

<u>CO_2 Separation from Air (400 ppm)</u>

$$\Delta G_{sep} = -\Delta G_{mix} = -\Delta S_{mix} = -nRT\sum_{i=1}^{N} x_i \ln(x_i)$$

$$= -1\ mol\ air \cdot 0.008314\,\frac{kJ}{mol \cdot K} \cdot 298\ K\big(0.0004 \cdot ln(0.0004) + 0.9996 \cdot ln(0.9996)\big)$$

$$= 8.74\,\frac{J}{mol\ air} \cdot 2500\ \frac{moles\ air}{mol\ CO_2}$$

$$\Delta G_{sep} = \mathbf{21.9}\,\frac{\boldsymbol{kJ}}{\boldsymbol{mol\ CO_2}}$$

<u>CO_2 Separation from Flue Gas (4.08%)</u>

$$\Delta G_{sep} = -\Delta G_{mix} = -\Delta S_{mix} = -nRT\sum_{i=1}^{N} x_i \ln(x_i)$$

$$= -1\ mol\ flue\ gas \cdot 0.008314\,\frac{kJ}{mol \cdot K} \cdot 298\ K\big(0.0408 \cdot ln(0.0408) + 0.9592 \cdot ln(0.9592)\big)$$

$$= 422\,\frac{J}{mol\ flue\ gas} \cdot 24.5\ \frac{moles\ flue\ gas}{mol\ CO_2}$$

$$\Delta G_{sep} = \mathbf{10.4}\,\frac{\boldsymbol{kJ}}{\boldsymbol{mol\ CO_2}}$$

References

1. Bains, P.; Psarras, P.; Wilcox, J. CO2 Capture from the Industry Sector. *Prog. Energy Combust. Sci.* **2017**, *63*, 146–172.

2. Hughes, S.; Zoelle, A.; Woods, M.; Henry, S.; Homsy, S.; Pidaparti, S.; Kuehn, N.; Hoffman, H.; Forrest, K.; Sheriff, A.; Fout, T.; Summers, W.; Herron, S. *Cost of Capturing CO2 from Industrial Sources*; DOE/NETL-2022/3319; National Energy Technology Laboratory (NETL), Pittsburgh, PA, Morgantown, WV, and Albany, OR (United States), 2022. https://doi.org/10.2172/1887586.
